# Real-World experience of interictal burden and treatment in migraine: a qualitative interview study

**DOI:** 10.1186/s10194-022-01429-5

**Published:** 2022-06-08

**Authors:** Siu Hing Lo, Katy Gallop, Timothy Smith, Lauren Powell, Karissa Johnston, Lena T. Hubig, Emma Williams, Vladimir Coric, Linda Harris, Gilbert L’Italien, Andrew J. Lloyd

**Affiliations:** 1 Acaster Lloyd Consulting Ltd, 8th Floor, Lacon House, 84 Theobalds Road, London, WC1X 8NL UK; 2 StudyMetrix Research LLC, 3862 Mexico Road, St. Peters, Missouri 63303 USA; 3 Broadstreet HEOR, 201 – 343 Railway Street, Vancouver, British Columbia V6A 1A4 Canada; 4 Biohaven Pharmaceuticals Inc, 215 Church Street, New Haven, Connecticut 06510 USA

**Keywords:** Migraine, Interictal burden, Health-related quality of life, Treatment experience, Qualitative

## Abstract

**Background:**

The debilitating nature of migraine attacks is widely established; however, less is known about how the interictal burden (i.e., how patients are affected in-between migraine episodes) of migraine impacts on patients’ health-related quality of life (HRQL). Acute and preventive treatments may lift the burden of the disease, but they often have unwanted side effects and limited effectiveness. The objective of this study was to understand the interictal burden of migraines, from the patient perspective, and to explore patient experience with migraine treatments.

**Methods:**

Participants (n=35) with a self-reported diagnosis of migraine were recruited in the US, UK and Canada, including a subgroup of patients who had taken calcitonin gene-related peptide monoclonal antibody (CGRP mAb) treatment for at least three months. Participants completed a background questionnaire, followed by a semi-structured interview via telephone or video call. The interviews explored patients’ migraine symptoms, perception of interictal burden and treatment experience. The interview transcripts were analysed using thematic analysis.

**Results:**

The most reported migraine symptom was migraine pain, followed by aura, sensory sensitivity and nausea. Most participants reported interictal impact on HRQL, lifestyle changes they made to avoid triggers or in anticipation of an attack, impacts on work, career, daily activities and relationships. Emotional impacts were reported by all participants, including anger, depression, anxiety and hopelessness. Many participants who took preventive treatments reported improvements in HRQL and functioning but still experienced breakthrough attacks. Among patients who took CGRP mAbs, participants noted varying consistency of treatment effectiveness between treatment administrations.

**Conclusion:**

This study detailed the additional HRQL impact of migraine in-between migraine attacks and described the unmet need for effective treatment options to prevent and mitigate migraine attacks.

**Supplementary Information:**

The online version contains supplementary material available at 10.1186/s10194-022-01429-5.

## Background

Migraine is a debilitating condition affecting more than one billion people worldwide and is characterised by recurrent episodes of severe head pain, which often occur concurrently with a number of other symptoms, including dizziness, irritability, nausea, sensitivity to light and sound, and problems with vision [1–3]. Migraines can be understood as a disease continuum, where the frequency, duration and severity may change over time.

Many treatments are available for those living with migraines. Acute migraine medication, including triptans, paracetamol and nonsteroidal anti-inflammatory drugs (NSAIDS), have been shown to reduce the symptoms and disease burden of migraines, but they do not prevent migraines from happening [4]. Antiepileptics, antidepressants and betablockers are used to reduce the frequency and severity of migraines, but some patients experience side effects and limited effectiveness [5]. Many patients report not being not satisfied with these older conventional treatments, and adherence can be poor [4, 5]. More recently, four calcitonin gene-related peptide monoclonal antibody (CGRP mAb) treatments have been approved for the prevention of chronic and episodic migraine [6–9]. These treatments inhibit the vascular CGRP receptors that are suspected to be the cause of migraine pain. Even with these treatments, many patients continue to need acute medications and some people have reported a waning of treatment effect [10, 11]. Most recently, a new oral CGRP receptor antagonist rimegepant has also been approved by the US Food and Drug Administration as both an acute and preventive treatment [12].

Research has demonstrated how migraines can cause substantial functional impairments and reduce patients’ health-related quality of life (HRQL) [13–16]. Migraine symptoms and its phases [17–19], as well as the impact of migraines on patients’ physical and cognitive ability, psychosocial and emotional well-being [15, 16, 18–22] have been extensively documented. However, in contrast to the substantial body of evidence regarding the specific impact of migraine attacks, relatively little is known about the interictal burden, or burden of disease between migraine episodes [15]. One study reported that interictal burden in migraine is common and both anxiety and avoidance (lifestyle compromise) can be interictal issues [23]. In this study, lost productive time was also associated with higher odds of interictal anxiety and avoidance. Another study aiming to synthesise the existing literature on the relationship between headache-attributed disability and lost productivity failed to identify studies exploring this relationship highlighting the dearth of research in this area [24]. Further, evidence suggests that interictal burden in migraine is a distinct effect of the disease that is not fully captured by other constructs such as depression and anxiety [25].

The present study aimed to gain a deeper understanding of the interictal burden of migraines, as well as explore patient experiences with acute and preventive migraine treatment. Qualitative interview methods were used to explore these issues with migraine patients in the United States (US), Canada and the United Kingdom (UK). The main focus of the interviews was to explore patient experiences of interictal burden of migraine. The experience of acute and preventive treatments was explored as a secondary study aim.

## Methods

### Sample

Eligible participants were adults with a self-reported diagnosis of migraine, who had not been treated with rimegepant, and were residents of the US, Canada or UK. The sample was designed to include a subgroup of participants who had experience of CGRP mAb therapy for a minimum duration of three months. The inclusion criteria for this subgroup were based on the clinical benefit needing to be assessed after at least three months of treatment for those receiving monthly administration of a CGRP mAb [26]. Participants were recruited by a specialist healthcare recruitment agency who identified potential participants through commercial database(s), social media, patient associations and physicians. Potential participants were provided with information about the study and if interested, completed a brief screener questionnaire to determine their eligibility (Additional file 1).

### Procedures

All participants provided informed consent and were asked to complete a brief background questionnaire including socio-demographics, current and past migraine treatment and details about their migraines. Each interview was tailored to the patient’s clinical and treatment history using the information provided in the questionnaire.

All interviews were conducted using a semi-structured interview guide by experienced qualitative interviewers. The interview guide was informed by a literature review of studies of migraine patients and included pre-dominantly open-ended questions with probes to elicit further details not spontaneously covered in the patient’s response (Additional file 2). The first part of the interview aimed to examine patients’ migraine symptoms and the impact on their daily life, emotional and social well-being. The interview then explored patients’ perceptions and awareness of any interictal burden of migraine (i.e., how patients are affected in-between migraine episodes). The last section of the interview focused on patients’ experiences and satisfaction with any treatments they had taken. For the subgroup of patients treated with CGRP mAbs, the interview also explored their experiences with waning of treatment effect.

Interviews were conducted via an online teleconference or video call and lasted between 30 – 80 minutes, with most lasting around 60 minutes. Participants received a study remuneration of $100 US/$125 Canada/£75 UK for their participation in the study. All interviews were audio-recorded and professionally transcribed verbatim and reviewed by the qualitative analyst for quality assurance.

### Analysis

The interview transcripts were analysed using thematic analysis. Thematic analysis is a qualitative method for identifying, analysing and reporting themes [27]. The analysis followed the procedures outlined by Braun and Clarke [27], including familiarisation, generation of codes, searching for themes, reviewing themes, defining the themes and reporting. The analysis aimed to identify concepts relating to the burden and impact of migraines, focusing on the interictal burden and participants’ acute and preventive treatment experience. Based on this, the concepts identified were grouped into themes. A coding framework was developed iteratively throughout the analysis. An initial coding framework was developed based on the interview questions and topics. Codes were then revised as the researchers read the transcripts, with new codes added as needed. Concept saturation was monitored during the analysis using a data saturation grid to ensure that all relevant concepts had been fully represented and followed the methodology suggested by Leidy and Vernon [28] to establish and document saturation. The saturation grid documents the number of individuals that identify a certain concept or category.

Data analysis was conducted using MAXQDA [29], a software tool that assists with organising qualitative data. Two team members independently coded a selection of interview transcripts initially. A third team member coded the same transcripts and a post-coding comparison and reconciliation occurred; all codes were compared, discussed, and reconciled whenever differences occur. When agreement between the coders was sufficient, two coders coded the remaining transcripts. The constant comparative method, an iterative coding approach moving between consecutive transcripts and new codes that emerged, was followed.

## Results

### Study sample

In total, 36 participants were screened and 35 participants completed an interview. Participant P002 withdrew from the study after screening as they no longer wished to take part. Participants were on average 40 years old, and the majority (91%) were female Table 1. Approximately half were employed full- or part-time and two-thirds had at least degree level education (69%).

**Table 1:** Study sample socio-demographic characteristics (*n*=35)

Sample characteristic	Statistic
Age	
Mean (SD)	40.0 (14.10)
Gender n (%)	
* Female*	32 (91%)
Employment status n (%)	
* Employed full time*	15 (43%)
* Employed part time*	3 (9%)
* Retired*	2 (6%)
* Unemployed*	4 (11%)
* Unable to work due to health problems*	4 (11%)
* Student*	4 (11%)
* Homemaker*	1 (3%)
* Other*	2 (6%)
Education n (%)	
* Primary education*	3 (9%)
* Secondary education*	8 (23%)
* Degree level or higher*	24 (69%)

A summary of the clinical data Table 2 showed that participants had an average of 12 migraine days per month in the last three months. The average age at diagnosis was 27 years, and over one-third of participants (37%) self-reported a diagnosis of chronic migraine. Half (51%) self-reported anxiety and one-third (34%) self-reported depression as comorbid conditions. Past and current treatments used by participants are summarised also. The high number of migraine days experienced indicates that these patients were not effectively treated. Also of interest is that these patients had clearly received many different treatments in the past to prevent their migraines, suggesting many of these treatments were ineffective or intolerable.

**Table 2:** Study sample clinical characteristics (*n*=35)

Sample characteristic	Statistic	
Migraine days per month (last three months) Mean (SD)	12.1 (8.0)	
Age at diagnosis Mean (SD)	26.5 (14.0)	
Type of migraine N (%)		
* Chronic migraine*	13 (37%)	
* Migraine with aura* ^a^	9 (26%)	
* Other migraine subtype* ^b^	8 (23%)	
* Don’t know*	9 (26%)	
Other long-term conditions		
* Anxiety*	18 (51%)	
* Bipolar disorder*	1 (3%)	
* Depression*	12 (34%)	
* Diabetes*	2 (6%)	
* Fibromyalgia*	4 (11%)	
* Hypertension*	3 (9%)	
* Post-traumatic stress disorder*	2 (6%)	
* Respiratory condition*	4 (11%)	
* Other*	6 (17%)	
* None*	11 (31%)	
Acute Treatments		
* Drug Type*	**Current/Most Recent Treatment**	**(All) Past Treatments**
* Pain relievers*	15 (43%)	14 (40%)
* Triptans*	18 (51%)	14 (40%)
* Anti-nausea drugs*	1 (3%)	1 (3%)
* Other*	4 (11%)	3 (9%)
Preventive Treatments		
* Drug Type*	**Current/Most Recent Treatment**	**(All) Past Treatments**
* Anti-epileptics*	8 (23%)	14 (40%)
* Anti-depressants*	2 (6%)	14 (40%)
* Beta-blockers*	2 (6%)	13 (37%)
* Neurotoxins (Botox)*	4 (11%)	6 (17%)
* Calcium channel blockers*	–	1 (3%)
* Angiotensin receptor blockers*	–	3 (9%)
* CGRP mAbs*	7 (20%) ^a^	8 (23%) ^b^
* Other*	3 (9%)	1 (3%)

^a^Includes classic migraine

^b^Includes migraine without aura, intractable migraine (with or without status migrainous), ocular migraine, hemiplegic migraine, vestibular migraine and (hyper) tension migraine

### Qualitative results

The saturation tables (Additional file 3) showed that no new codes were added in the final 13 interviews, indicating that concept saturation was reached. The following sections will summarise the findings relating to participants’ experience with migraine and the impact of migraines on their HRQL, focusing on the interictal burden of migraines and how participants compare themselves to others without migraine and participants’ experience of treatment.

#### Migraine symptoms

Participants were asked about the symptoms they experienced during or immediately after migraine episodes. Some common symptoms (pain, nausea, light/sound sensitivity, blurred/affected vision) were often probed by interviewers if not reported spontaneously, however due to the semi-structured approach used, not all participants were asked all probe questions. The most commonly reported symptoms (prompted or unprompted) included pain, aura, affected vision, light or sound sensitivity, as well as nausea and dizziness Table 3.

**Table 3:** Symptoms experienced during a migraine (*n*=35)

Symptom Type	Number of patients self-reporting symptom (prompted or unprompted)^a^
Pain	35
Light/sound sensitivity	33
Nausea	26
Other symptom type^b^	20
Blurred/affected vision	17
Aura	10
Cognition problems	10
Dizziness	10
Tiredness	10
Stiff neck	7
Lack of awareness/disorientation	4
Smell sensitivity	4
Feeling numb	3
Slurred speech/trouble speaking	3

^a^All self-reported symptoms; participants were able to report multiple related or unrelated symptoms

^b^Such as vertigo, head pressure, heavy head, feeling too hot or too cold, body distortion, spasms, and increased heart rate

Migraine pain was a significant focus for all participants, with participants describing the intensity and location of the pain experienced. In addition to pain, a number of participants experienced aura with their migraines, manifesting predominantly for participants as seeing zigzag lines, flashing lights or black spots in their vision. Further, many participants, including those who experience aura and those who do not, also reported blurred vision or even loss of sight as a result of their migraines. Participants reported heightened sensitivity to light and sound, and many talked about avoiding exposure to any light or sound during a migraine. A few participants also reported heightened sensitivity to certain smells during a migraine, such as cigarette smoke and strong perfume. Many participants experienced nausea during a migraine which could lead to dizziness and vomiting. A few participants also described cognitive issues or brain fog, feelings of tiredness or numbness, slurred speech or trouble speaking, frequent bowel movements, disorientation as well as a stiff neck and heavy head.

Many participants reported experiencing symptoms immediately following a migraine, (referred to as postdrome symptoms). These included tiredness and fatigue or feeling hungover with a level of brain fog. Beyond fatigue and feeling hungover, participants described lingering and residual symptoms of their migraine attacks that remain after the migraine has gone away. This included sensitivity to light, nausea, or a slight headache, typically occurring the day after a migraine.

#### Interictal burden

Participants discussed the interictal burden of migraine, specifically the ways migraine has affected their behaviour, career, daily activities and social functioning, and the psychological burden of migraine between attacks.

##### Behaviour and lifestyle changes

Many participants discussed changes they had made to their daily routines due to migraines, including eating regular meals and adjustments to their sleep schedule. All participants avoided certain migraine triggers such as specific foods and drinks as well as sensory stimuli. These included flashing lights, fluorescent lights and looking at a computer screen for long periods of time as well as sound and smell sensitivity such as loud music and strong perfume. Some stated they always carried acute medication in anticipation of another migraine attack.

Participants also described lifestyle changes that they had made. This included the avoidance of stress and changes to physical activity. Some participants reported increased exercise levels in efforts to reduce migraines whilst others avoided physical exertion and gave up certain sports. Some participants were concerned their lack of movement as a result of migraines had affected their general physical health. The prolonged sedentary approach to managing their migraine symptoms had led to an unhealthier lifestyle where they gained weight or lost a level of fitness they had previously achieved, as explained by one participant:

*“I guess in terms of my physical health, my sort of fitness, it’s impacted on that. Um, in that I feel that I’ve put on weight and I’m not as fit as I used to be…Um I think sometimes because I overeat because of feeling so low about the migraines and sometimes eating does give temporary relief…[me] not being able to exercise as much as I would like to.” – P019, chronic migraine, female, age 45, UK*

##### Changes to work and daily activities

The impact on work or education was one of the most common and significant issues reported by participants. Many people described how work or school was often affected on a day-to-day basis. Some participants believed that the burden of their migraines meant they lost out on promotion opportunities in their career. Some people had to stop working altogether because their migraines were becoming too invasive and debilitating. Migraines also disrupted daily routines and activities and work around the house. Participants described how activities requiring concentration were particularly affected.

Many people described how they had made changes to their work due to their migraines. Changes included reducing hours at work, quitting work, planning work or study around migraine attacks, and avoiding overtime. People described efforts to avoid stress related to work. Others had made adjustments to the work environment, such as specialist equipment and sitting somewhere in the office with less light stimuli or wearing sunglasses. Furthermore, two participants did not work because they did not feel reliable enough and one had been asked to leave her business because of her migraines.

*“I had a successful art business, um, five years’ ago. So that’s gone be-because I can’t work on it. I used to run a gallery with three other artists, the four of us used to run it as a cooperative. They asked me to leave because of the migraine” – P016, migraine with aura, female, age 39, UK*

##### Psychological impact and burden

All participants reported an emotional impact from their migraines, including irritation or anger, anxiety or worry, as well as feelings of vulnerability. Many also raised feelings of sadness and depression because of their migraines, where they felt hopeless about their condition and the prospect of having to endure migraines for the rest of their lives. Many also reported feelings of loneliness and isolation. Participants described how they would routinely be alone at home instead of spending time with loved ones. While many participants shared feelings of isolation, a small minority also discussed how it had led to suicidal ideation.

Many of the participants said that when not experiencing a migraine, they worried or were anxious about when their next migraine attack would be. Several experienced a constant underlying anxiety or felt constantly “on edge” [P006]. For many, this worry stems from whether their next migraine will impact upcoming work, plans or activities, and any guilt associated with cancelling plans. In one participant’s words:

*“My closest friends will make plans with me and then you know, they’ll insist “If you don’t- if you have a headache, don’t worry, you don’t need to come” but then I still feel guilty, I feel like I’m very unreliable. And you know, often times I just won’t make any plans because I don't know if I’m gonna be able to do it” – P032, migraine type unknown, female, age 57, Canada*

A few participants also mentioned a negative impact on their confidence and self-esteem, and feelings of lack of control or uncertainty. Other emotional impacts reported when not experiencing a migraine included hopelessness, fear and frustration. One participant described how migraines impacted their confidence:

*“It does knock your confidence because you think, ‘Oh well they probably think I’m just using it as an excuse,’ or like I’m not working as hard today because I’ve got this, and they’re thinking, ‘Oh you’re just being lazy’” – P013, migraine with aura, female, age 28, UK*

Despite many reporting negative impacts on their emotional wellbeing, they also felt they were happy when they didn’t have a migraine. One participant describes the absence of a migraine on their mood as follows:

*“My mood improves on the days when I haven’t got a migraine. I think you know, there’s always a bit of anxiety there, …but in myself, I do feel much happier yeah” – P028, chronic migraine, female, age 57, UK*

Participants discussed what their life is like in comparison to people who don’t experience migraines. Many of the participants said that their daily activities differed vastly, such as how they spend their days before, during and after migraines, planning daily and future activities and productivity. In one participant’s own words:

*“They are able to do, live their best life. Do things, anything that they want to and I’m basically not able to do that because when I’m dealing with those migraines, it’s like it’s time for you to sit down or lay down and be quiet” – P008, chronic migraine, male, age 40, male, US*

##### Social functioning

Participants reported that their migraines could impact or strain their relationships. Many participants discussed how they had cancelled or postponed plans to see their family and friends as a result of migraines whilst others stopped making any social arrangements due to the uncertainty of their condition. Some participants reported being moody or rude to their loved ones during a migraine, which they felt had damaged their relationships. Others described feeling like a physical and financial burden due to the impacts of their condition. A large number of participants discussed people in their lives, including friends and family, who did not have an awareness or accurate understanding of the severity of migraines, and how this would particularly affect relationships.

Although migraines could negatively impact participants’ relationships, many reported that they received support from friends and family. This included both practical support, such as help with household chores, as well as emotional support, for example understanding the impacts of migraines on the individual.

Many said that they were unable to plan or commit to activities including days out, social events or holidays because they expected a migraine to happen. One participant described always having to be prepared for their next migraine:

*“It’s always there, like the little demon that’s always ready to jump out and attack you, like I always have to like be ready for it, you know?” - P001, chronic migraine, female, age 41, US*

Some also stated similar changes to their social life including avoiding seeing friends and family, avoiding going out with friends when others would be drinking alcohol, avoiding any events that would lead to staying out late, leaving social gatherings early, and avoiding places such as nightclubs or cinemas.

#### Treatment experiences and preferences

##### Experience with acute migraine medication

Many participants reported that their acute medication for treating migraine symptoms was less effective than they hoped or expected. For some it did not reduce the severity of symptoms experienced at all or as much as they would like it to. Some participants reported that their acute medication reduced the severity of their symptoms to a manageable level, allowing them to continue with their daily activities. Others reported that their acute medication could reduce the duration of a migraine or prevent a headache from progressing into a migraine if taken when symptoms were first experienced. However, many participants described how these treatment benefits could be inconsistent. Only a few reported that their acute medication relieved their migraines completely. Others only got temporary (one to three hours) symptom relief. Some people thought treatment was slow to work and wanted instant relief, whereas others were happy if it worked within two hours. This comment was typical:

*“I would still have a migraine but it’s more tolerable.” – P023, migraine type unknown, female, age 27, US*

Many different side effects were reported, with tiredness or drowsiness the most commonly reported side effect. Other problems attributed to treatment included nausea, gastrointestinal issues, low mood and brain fog. Some people reported that they had changed treatments in the past because of side effects. However, for some people their acute medication also had benefits beyond symptom relief. Having reliable acute medication reduced people’s anxiety about migraines and for some it also meant that they were able to continue working even if they got a migraine.

##### Experience with preventive migraine medication

Among participants who took preventive medication, many reported that the frequency of their migraines had decreased. For one participant, the fact that they now had days without headaches was a “game-changer” [P001]. Since initiating preventive treatment some participants experienced shorter duration or less severe migraines. A few stated they used acute medication less due to the efficacy of their preventive medication. Others expressed disappointment with still experiencing breakthrough migraines despite using preventive medication. For side effects, participants reported tiredness or drowsiness, nausea, gastrointestinal issues, weight gain and brain fog.

Some participants reported that their daily activity levels were better since taking preventive treatment. For one participant it “gave me my life back” [P014]. A few participants reported better emotional well-being and less worry as it had reduced their migraines or felt more normal. However, some had stopped taking preventive medication due to the impact of side effects on their work and daily life.

A sub-group of 12 twelve patients had used CGRP mAb treatments to prevent migraine. Most felt that the treatment had led to a large reduction in the number of migraines they experienced. One participant now had “crystal clear days” with no form of headache and described their treatment as a miracle drug [P015]. Another participant described their treatment as:

*“…really good in reducing my daily levels of pain, my headache. I was having a lot of severe pain when I started it. I got down to only a few severe pain days a month, and it reduced my migraines from around 16 a month down to about the 6 to 8.” – P016, migraine with aura, female, age 39, UK*

Others reported less benefit – but most agreed that the severity of migraines was reduced. Adverse events reported from CGRP mAb treatments included injection site reactions, insomnia, weight gain, brain fog, muscle or joint pain and gastrointestinal issues; and some had stopped treatment due to the adverse events. Some participants reported that CGRP mAb treatments improved their daily activities and work, with treatment making things more manageable, and meant they were not missing out on social plans and were able to exercise more.

##### Wearing off effects of injectable preventive medication

Among participants with experience of injectable preventive medication (CGRP mAbs or Botox), the consistency of treatment effectiveness between treatment doses varied. Most who had taken a  CGRP mAb treatment had not noticed the effect wearing off within the treatment cycle, but a few had previously noticed the effect waning between injections until their dose was increased and a few reported that the effect only lasted one or two weeks after each dose. One participant discussed the reduced severity of migraines wearing off towards the end of the treatment cycle, when their migraines were “horrible” [P018]. Another participant described the need to increase the dose to avoid wearing off effects:

*“Um, and I also suspect that, um, my body had started to identify the [….] in my system, because when I first started it right back to the beginning it would sort of wear off about a week before I was due an injection, and then after I’d gone up to the higher dose it stopped doing that and it was lasting the entire four weeks.” – P016, female, age 39, UK*

Similarly, some participants with experience of Botox treatment also found that the benefits wore off towards the end of the treatment cycle.

## Discussion

Participants in this study described a range of migraine symptoms and different experiences with migraine frequency, duration, and severity, consistent with previous research [16, 18, 19]. While the experience of migraine attacks varied between participants, there were common themes with respect to the HRQL domains impacted by migraines. Participants discussed the significant impact their migraines had on their daily life and activities, work/career, relationships/family, social life and hobbies, sleep, as well as its emotional impact.

Previous research has demonstrated that individuals with migraine experience interictal burden [23, 25]; however, to our knowledge this is the first study to qualitatively explore the nature and impact of the interictal burden. The results showed that this burden includes feelings of being unable to plan, feeling unreliable (in terms of planned activities and seeing people), needing adaptations to daily routines, lifestyle changes, reducing or stopping work, avoiding social or family activities and feeling anxious about the next migraine attack. These findings highlight the burden of migraines for people not only in terms of the impact experienced during a migraine episode but also the overall burden experienced between episodes. The study describes the patient experience between migraines and the consequences for peoples’ relationships, activities and work, and emotional wellbeing. It is clear that the impact of migraines on patients is not restricted to the episodes themselves, and comparative effectiveness research should consider hangover effects of migraines (on usual activities, symptoms and HRQL) as well as other interictal phenomena such as the anxiety reported in this study.

The results from the interviews also highlighted the huge variability between patients in terms of their experience of migraines, the impact on their life and their perceptions regarding the effectiveness of treatments. Some patients reported experiencing episodes almost every day and any reduction in that was considered very beneficial. As the frequency of migraines was reduced with therapy, people reported valuing the time between episodes more. Participants knew that they would still experience migraines, but symptom-free days were highly valued. Other participants described how preventive therapies reduced the severity or duration of migraines which made the experience more manageable. Except for a few cases, there was still a large unmet need in this patient group in terms of preventing migraines and treating them when they did occur. Some evidence also emerged that the effectiveness of preventive treatments may reduce over time. More evidence is needed to formally assess this [11], but it also highlights that there is a level of unmet need in this patient group, as discussed in previous literature [30, 31].

Some limitations in this research should be considered. In line with most qualitative research, this study recruited a relatively small sample of people, and those with a higher disease burden and experience of CGRP mAb treatments were purposively oversampled to enable the study to explore the widest possible range of migraine patient experiences. The intention here is not to determine the proportion of people with each migraine symptom or different views on aspects of migraine care. Instead, the aim is to try to describe the range of experience associated with migraines and interictal burden. Concept saturation was assessed to describe the extent to which the data captured the range of experience. The benefit of this approach is the richness of the data which illustrates and describes how migraines affect people. Using this approach on this sample of participants we reached saturation on the main concepts of interest. One limitation of this approach, however, is that it does not lend itself to a rigorous exploration of differences by migraine type or in terms of other sub-groups (e.g., by study country/healthcare system) that may have contributed to the heterogeneity in patient experiences observed in this study. These differences would be better quantified in future quantitative research. There are other limitations that are relevant here too. The study was carried out during the COVID-19 pandemic, and all interviews were conducted using video conferencing software. It is possible that the use of video software impeded communication between the interviewer and participant and affected people’s ability and willingness to participate. Face to face interviews were impossible at this time and so video interviews were considered the best option [32, 33]. As only English-speaking people in the US, Canada and the UK were included in the study, our results may not be consistent with the experience of patients in other populations. In addition, our sample had a high proportion of women (91%), which is slightly more than we would anticipate based on epidemiological data [34]. A different sample of people may produce different data, but we believe that the major themes would be consistent. The sample also consisted of participants who had self-reported a migraine diagnosis received from a medical doctor, and as such their diagnosis was not independently verified.

## Conclusions

This study explored the manifold aspects of the migraine interictal burden. Patients described serious impacts on their HRQL, work and relationships between migraine episodes, adding to their overall burden of disease. These findings highlight that, despite many treatment options, there is substantial unmet need for effective treatments, which not only eliminate or reduce the HRQL impacts experienced during a migraine episode but also mitigate the overall burden experienced my migraine patients.

## Supplementary Information

**Additional file 1:** Screening questionnaire. The screening questionnaire determined an individual’s eligibility to take part in the study based on the inclusion and exclusion criteria.

**Additional file 2:** Interview guide. The interview guide was used by experienced qualitative interviewers to lead the discussion and included predominately open-ended questions with prompts to elicit further details not spontaneously covered in the patient response.

**Additional file 3:** Saturation tables. The saturation tables document the individuals that identify a certain concept or category.

## Data Availability

The datasets used and/or analysed during the current study are available from the corresponding author on reasonable request.

## Abbreviations

CGRP: Calcitonin gene-related peptide
HRQL: Health-related quality of life
mAb: monoclonal antibody
