# Supplementary material for: Real-World experience of interictal burden and treatment in migraine: a qualitative interview study
Source: J Headache Pain. 2022 Jun 8;23(1):65. doi: 10.1186/s10194-022-01429-5 (PMC9174626; doi:10.1186/s10194-022-01429-5)
Supplement: Supplementary file 1 — Additional file 1: Screening questionnaire. The screening questionnaire determined an individual’s eligibility to take part in the study based on the inclusion and exclusion criteria. [file 10194_2022_1429_MOESM1_ESM.docx]

Additional file 1

Screening questionnaire

1. What is your age?

|_|_|

**[SCREEN OUT IF UNDER 18 YEARS]**

1. Have you consulted a doctor or other healthcare provider as a result of any of the following symptoms or conditions in the past twelve months? *Please select all that apply.*
   1. Coughing or sore throat
   2. Fever
   3. Nausea or vomiting
   4. Headache
   5. Dizziness
   6. Migraine **[SCREEN OUT IF NOT SELECTED]**
2. Have you been diagnosed by a medical doctor with migraine?
   1. Yes
   2. No **[SCREEN OUT]**
   3. Don’t know **[SCREEN OUT]**
3. **How many days** have you had a **migraine** in the last twelve months?

|_|_|_| migraine days in the past year **[SCREEN OUT IF <2 MIGRAINES]**

1. How often have you had a migraine in the last month?
   1. More than 15 days a month
   2. At least once a month but less than 15 days a month
   3. Less than once a month

**[SOFT QUOTAS: AIMING FOR MIX ACROSS RESPONSE OPTIONS]**

1. Have you ever taken any of the following medication for your migraines?
   1. Aimovig (erenumab)
   2. AJOVY (fremanezumab)
   3. Emgality (galcanezumab)
   4. NURTEC ODT (rimegepant) **[SCREEN OUT]**
   5. Vyepti (eptinezumab)
   6. None of the above
2. How long have you taken **[INSERT Q6]?**

|_|_| months OR |_|_| years

**[8-10 PATIENTS TAKEN [INSERT Q6] FOR A MINIMUM OF 3 MONTHS]**
