# Supplementary material for: Real-World experience of interictal burden and treatment in migraine: a qualitative interview study
Source: J Headache Pain. 2022 Jun 8;23(1):65. doi: 10.1186/s10194-022-01429-5 (PMC9174626; doi:10.1186/s10194-022-01429-5)
Supplement: Supplementary file 2 — Additional file 2: Interview guide. The interview guide was used by experienced qualitative interviewers to lead the discussion and included predominately open-ended questions with prompts to elicit further details not spontaneously covered in the patient response. [file 10194_2022_1429_MOESM2_ESM.docx]

Additional file 2

Interview guide

**All moderator instructions are in red and probes are in grey – DO NOT READ TO THE PARTICIPANT**

**Only use probes once participants have had an opportunity to answer questions unprompted and only if appropriate and relevant; you do not have to use all probes.**

**Throughout the interview:**

1. **Use the responses to the background questionnaire to make questions relevant to the participant**
2. **Only ask questions/use probes where appropriate, considering what has already been discussed.**
3. **Important questions and probes are in bold**

## Introduction to the project **[~2 minutes]**

Good (morning/afternoon/evening), my name is [insert name] and I am a researcher at Acaster Lloyd Consultancy Ltd. Thank you for taking time to participate in this interview. We are interested in hearing about your experiences with migraine, the impact of migraines on your quality of life, as well as your experiences with and views on migraine treatments.

The objective of the study is to better understand people’s experiences with migraine as well as people’s views on treatments for migraine. Your participation is voluntary, so you can stop participating at any time without giving us a reason. We expect this conversation to last up to an hour.

I will be recording this interview, so we don’t miss any of your comments. Do you agree for the interview to be recorded? **[If yes]** Okay, thank you. I’ll start recording now.

**If the participant does not wish for the interview to be recorded, the interviewer should not proceed with the interview.**

**START RECORDING**

Today is [DATE]. This is participant ID [INSERT ID NUMBER].

Before we start can I confirm a few things:

- I am not a medical doctor, so I am not qualified to give medical advice. If you have any questions about migraine as a result of our conversation today, I advise you to follow up with your regular doctor.
- There are no right or wrong answers, we understand that everyone has different experiences, and we are interested in what you have to say.
- Please speak loudly enough to be heard for the recording.
- To let you know, I will be taking notes during the interview so please know, that if I am quiet and typing, I am listening attentively to your responses.
- Do you have any questions before we begin?

## Section A: Background **[~5 minutes]**

To start off, I would like to get an idea about who you are and your life.

1. Please could you tell us about yourself?

Age, work/education/day-to-day activities, hobbies, family (children)

1. You stated in the questionnaire that you were diagnosed [STATE WHEN DIAGNOSED]. Can you tell me about what led to your diagnosis with migraine?

Who diagnosed (GP or specialist)

1. ***If mentioned ‘chronic migraine’ or ‘episodic migraine’*: You stated in the questionnaire that you have been diagnosed with [STATE TYPE OF MIGRAINE].** What does it mean to you to have **[STATE TYPE OF MIGRAINE]**?

***If don’t know migraine type:* [ASK QUESTIONS TO HELP IDENTIFY TYPE OF MIGRAINE BASED ON RESPONSES TO BACKGROUND QUESTIONNAIRE]**

**Migraine with or without aura, triggers of migraine, frequency of migraine**

**Changes in migraine type over time: any switch from episodic to chronic migraine and vice versa**

## Section B: Symptoms and Impacts of Migraine on Daily life **[~20 minutes]**

The next section involves questions about your life when you have a migraine.

1. **Tell me about your migraine symptoms**

**Symptom type – pain, nausea, light/sound sensitivity, blurred/affected vision^1,2^**

**Symptom frequency – on average/typically, variability, any patterns/predictability**

**Symptom duration – on average, variability**

**Symptom severity – mild, moderate, severe and why, variability**

Sequence of symptoms – what symptoms are experienced first, what last

Symptom variability – same or different symptoms across migraine attacks

1. **Are there any things that make your migraine symptoms worse?**

**Triggers prior to attack vs. things that make it worse during attack**

Impact on symptom severity, duration and frequency

1. **Tell me about how your daily life and activities are affected when you have a migraine**

**Work/education, days off work/school, limited productivity, impacts on career**

**Housework: household chores^1–3^, preparing food; *if applicable*: caring for children?^4^**

**Activities requiring concentration/thinking clearly/staying alert?^1–3^**

Getting out of bed, ready for the day^1–3^

Keeping daily routine^1,3^

Activities requiring physical effort^3^

General mobility/activity, needing to lie down, weakness, fatigue^3^

Specific movements: moving head or body, bending over, standing up for long periods or walking^3^

1. Do your migraines impact your relationships with other people when you have a migraine? If so, how?

Activities and social interactions with family, friends, and co-workers^3,5^

Being around other people^3^

Understanding from others

Relationship with partner

1. Do your migraines impact your social life and hobbies when you have a migraine? If so, how?

Activities outside the home^3^

Difficulties reading

Leisure activities^3^

1. Do your migraines impact on your sleep when you have a migraine? If so, how?

Ability to sleep, needing more sleep, sleeping during the day^1^

1. **Do your migraines impact you emotionally? If so, how?**

**Irritation/frustration^1–3,5^**

**Anxiety/worry^3,5^**

**Disappointed/sad/depressed^3^**

**Lack of control^5^**

Ashamed/embarrassed^3,5^

Guilt/feeling like a burden on others^3^

1. Do you experience any symptoms or impacts on your daily life immediately after you have had a migraine?  If so, can you describe these symptoms and impacts? How long do you experience these?

Feeling like you are hungover

Euphoria/relief that migraine is over

Residual symptoms from migraine attacks (e.g. residual head pain)

Tiredness, fatigue, decrease in energy, weakness

Nausea, dizziness, light-headedness

Sleepiness

Concentration problems

Soreness, neck tension, stiffness

Loss of appetite

Impacts on daily activities: work, housework, leisure activities, family and social life

## Section C: Life while not having a migraine or headache **[~10 minutes]**

In the next section, I will ask you questions about your life on days when you do not have a migraine.

1. **a. Are there any things you avoid or do to prevent migraines when you do not have a migraine? *If yes*: can you describe what do you avoid or do?**

**b. Have you made any lifestyle changes because of migraines? *If yes*: can you describe these changes?**

**Changes to daily routines^5^**

**Changes to physical exercise, hobbies**

**Foods/drinks** – aged cheese, salty and processed foods, food additives, alcohol, caffeine

**Sensory stimuli** – bright lights, sun glare, loud sounds, strong smells

**Changes to work**

**Other lifestyle changes**

Stress

Sleep changes

Physical exertion, incl. sexual activity

Preventative medication

Care-giving responsibilities

Avoidance of migraine triggers

1. **Do you experience any impact of migraine on your life when you do not have a migraine?  *If yes*: Can you describe how your life is affected? What in your life is affected most?**

**Doing less/reduced activity levels**

**Feelings of worry/shame/embarrassment/lack of control**

**Avoiding migraine triggers**

**Anticipation of experiencing another migraine attack**

**Fear that particular tasks/important events will not go well if you have a migraine attack**

1. **Do migraines impact your emotional well-being when you do not have a migraine?**

***If yes*: Can you describe how you are affected emotionally? To what extent?**

**Worry about next migraine attack**

**Worry about migraine triggers**

**Worry about certain activities in case of having a migraine**

**Impact on confidence, self-esteem, sense of well-being**

**Embarrassment/shame**

**Feeling fed up or irritated**

**Feelings of lack of control/uncertainty**

1. **How do you feel your life compares to that of people without migraine?**

**Daily activities**

***If applicable*: impacts on career/work/school**

**Family life**

**Social life**

**Hobbies and leisure activities**

**Doing or not doing things to avoid migraine triggers**

1. Are there any other impacts of migraine that we haven’t discussed yet?

## Section D: Views and preferences for treatment **[~20-25 minutes]**

We will now move on to your experiences with and views on migraine treatments.

1. ***For all current acute treatments*:** You stated in the questionnaire that you use(d) **[list acute medications]** for your migraine attacks.

**a. *If multiple current acute treatments:* Could you describe when you would typically take these medications [for each current acute treatment]? Why?** How long have you used these medications?

**Impact on symptoms experienced, severity and duration after medication**

***If one current acute treatment****:* **Do/did you always take it when you have/had a migraine attack? Why?** How long have you used **[acute medication]**?

**b.** Could you tell me about your experience with **[acute medication(s)]**?

**c. Tell me about how satisfied you are/were with [acute medication(s)].**

**Reliability of medication^7^**

**Effectiveness of medication^7^**

**Speed of relief^7^**

**Duration of effect^7^**

**Side effects^6^**

**Mode of administration^7^**

**d. Was your life in between migraine attacks affected in any way by being able to take [acute medication] during a migraine attack? How?**

**Reduction in worry in between attacks**

**Relationship between effectiveness/speed of relief, and worry between attacks**

1. ***If taken more than one acute treatment*: What is your preferred treatment for when you have a migraine attack?**

**a. What do you like about it? How does it differ to other treatments?**

b. Is there anything you would change about it? Can it be improved? *If yes*: how?

Reliability of medication working^7^

Effectiveness of medication^7^

Speed of relief^7^

Duration of effect^7^

Side effects^6^

Mode of administration^7^

1. ***For all current preventative treatments other than mAbs*:** You stated in the questionnaire that you use(d) **[list preventative medication(s)]** for your migraine.
   1. How long have you used **[each preventative medication]**? **How regularly are/were you taking [each preventative medication]? Why?**

**Impact on symptoms experienced, severity and duration after medication**

**b**. Could you tell me about your experience with **[each preventative medication]**?

**c. Tell me about how satisfied you are/were with [each preventative medication].**

**Effectiveness/reliability of it preventing migraines^9^**

**Duration of preventative effect**

**Length of time before any preventative effect is noticed**

**Side effects^8^**

**Mode of administration^10^ and treatment schedule; any flexibility in dosing/schedule**

**Storage of medication, preparation and administration time**

**Half-life**

**d. Was your life in between migraine attacks affected in any way by taking [preventative medication]? How?**

**Reduction in worry in between attacks**

**Relationship between effectiveness/reliability in prevention, and worry between attacks**

**e. Which aspects of treatment effectiveness are relatively more/less important?**

**Reduction of number of migraine days**

**Reducing the severity of migraines**

**Whether the treatment effectiveness wears off before the next dose**

# ***For all mAbs treatments only*: You stated in the questionnaire that you use(d) [mAbs medication] for your migraine.**

# **a. How often do you have an injection?**

# **b. Tell me about your experience with [mAbs medication].**

**c. Tell me about how satisfied you are/were with [mAbs medication].**

**Effectiveness/reliability of it preventing migraines^9^**

**Duration of preventative effect**

**Length of time before any preventative effect is noticed**

**Side effects^8^**

**Mode of administration^10^ and treatment schedule; any flexibility in dosing/schedule**

**Storage of medication, preparation and administration time**

**Half-life**

**d. Treatment experience from one dose until next dose**

**e. Any experiences with treatment effects diminishing before next dose**

**f. Was your life in between migraine attacks affected in any way by [mAbs medication]? How?**

**Reduction in worry in between attacks**

**Relationship between effectiveness/reliability in prevention, and worry between attacks**

1. ***For all mAbs treatments only*: How long have you been on [mAbs medication]? Has your experience with the treatment changed since you started taking it?**

***If yes*: can you tell me how it has changed?**

1. ***If taken any preventative treatments and experience breakthrough migraines*: You mentioned you experience migraines when you are/were on preventative treatment.**
   1. **Do these differ from migraines that you experienced before you took [preventative medication]? How?**
   2. **Do you use [acute medication] to treat these migraines? What do you use?**
   3. Do these migraines affect how you feel about your **[preventative medication]**? How?

**Type of symptoms**

**Symptom severity**

**Predictability of any migraines experienced during the course of any preventative treatment**

**Migraines occurring in relation to when the dose was administered**

**Expectations of preventative treatment**

**Disappointment with breakthrough migraines**

1. ***If taken more than one preventative treatment*: what is your preferred treatment for prevention of migraine?**

**a. What do you like about it? How does it differ to other treatments?**

**b. Is there anything you would change about it? Can it be improved? *If yes*: how?**

**Effectiveness/reliability of it preventing migraines^9^**

**Duration of effect**

**Length of time before any preventative effect is noticed**

**Side effects^8^**

**Mode of administration^10^ and treatment schedule; any flexibility in dosing/schedule**

**Storage of medication, preparation and administration time**

**Half-life**

**c. Which side effects are relatively more/less important?**

**Type, severity or duration of side effect**

1. ***If more than one current and/or past treatment***: Do you ever take more than one medication at a time? If so, can you tell me about your experience with taking medications together?

Side effects, drug-drug interaction

Convenience or inconvenience of taking multiple treatments at the same time

1. ***If experience with acute and preventative treatments*: Would you like to be able to take one medication for acute attacks and prevention? *If yes*: can you explain why? Would it be important to you? *If yes*: can you explain why?**

Relative importance of being able to take a treatment for both acute and preventative purposes

1. Tell me about anything else you do to manage your migraine attacks?

Doctor visits, hospital visits

Alternative therapies

1. **Would you like to try any treatments that you have not yet been prescribed? Why?**

**Awareness of new medications**

1. What are your wishes and hopes for future migraine treatment?

Effectiveness/reliability

Side effects

Mode of administration and treatment schedule

Flexibility of when to take medication

**Thank you for your help with this research.**

**We really appreciate the time you have taken to participate in this study.**

References

1. Speck RM, Collins EM, Lombard L, Ayer DW. A Qualitative Study to Assess the Content Validity of the 24‐Hour Migraine Quality of Life Questionnaire in Patients with Migraine. *Headache J Head Face Pain*. 2020;60(9):1982-1994. doi:10.1111/head.13915

2. Houts CR, Wirth RJ, McGinley JS, et al. Content Validity of HIT‐6 as a Measure of Headache Impact in People With Migraine: A Narrative Review. *Headache J Head Face Pain*. 2020;60(1):28-39. doi:10.1111/head.13701

3. Mannix S, Skalicky A, Buse DC, et al. Measuring the impact of migraine for evaluating outcomes of preventive treatments for migraine headaches. *Health Qual Life Outcomes*. 2016;14(1):143. doi:10.1186/s12955-016-0542-3

4. Leonardi M, Raggi A. A narrative review on the burden of migraine: when the burden is the impact on people’s life. *J Headache Pain*. 2019;20(1):41. doi:10.1186/s10194-019-0993-0

5. Palacios-Ceña D, Neira-Martín B, Silva-Hernández L, et al. Living with chronic migraine: a qualitative study on female patients’ perspectives from a specialised headache clinic in Spain. *BMJ Open*. 2017;7(8):e017851. doi:10.1136/bmjopen-2017-017851

6. Sacco P, Shah S, Jackson L, Price M, Bensink M, Bernstein C. Beyond frequency alone-exploring what meaningful improvement means in the treatment of migraine: Focus groups in patients and health care providers. *Headache Conf 62nd Annu Sci Meet Am Headache Soc*. 2020;60((Supplement 1)):130.

7. Shrewsbury SB, Ailani J, Ray S, Aurora SK, Hoekman J. Impact and burden of episodic, acute migraine: A patient experience study. *Headache Conf 62nd Annu Sci Meet Am Headache Soc*. 2020;60((Supplement 1)):71. doi:10.1111/head.13854

8. Scheffler A, Messel O, Wurthmann S, et al. Erenumab in highly therapy-refractory migraine patients: First German real-world evidence. *J Headache Pain*. 2020;21(1):84. doi:10.1186/s10194-020-01151-0

9. Ford JH, Jackson J, Milligan G, Cotton S, Ahl J, Aurora SK. A Real-World Analysis of Migraine: A Cross-Sectional Study of Disease Burden and Treatment Patterns. *Headache J Head Face Pain*. 2017;57(10):1532-1544. doi:10.1111/head.13202

10. Matza LS, Deger KA, Vo P, Maniyar F, Goadsby PJ. Health state utilities associated with attributes of migraine preventive treatments based on patient and general population preferences. *Qual Life Res*. 2019;28(9):2359-2372. doi:10.1007/s11136-019-02163-3
