# Supplementary figures and images for: Real-World experience of interictal burden and treatment in migraine: a qualitative interview study
Source: J Headache Pain. 2022 Jun 8;23(1):65. doi: 10.1186/s10194-022-01429-5 (PMC9174626; doi:10.1186/s10194-022-01429-5)

Additional file 3

Saturation tables


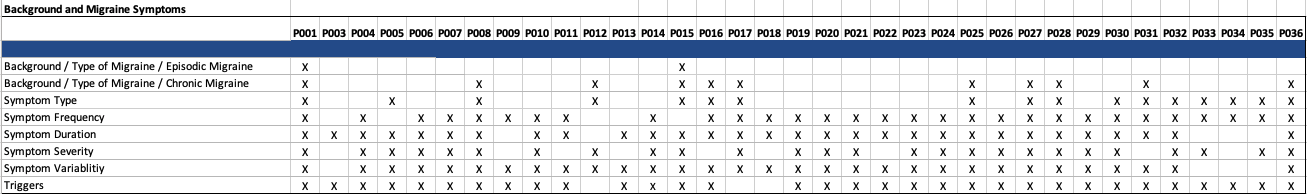


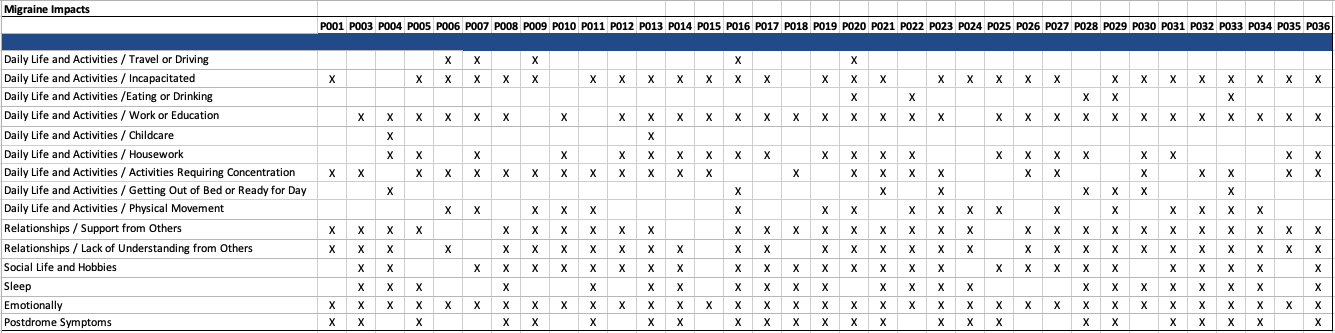


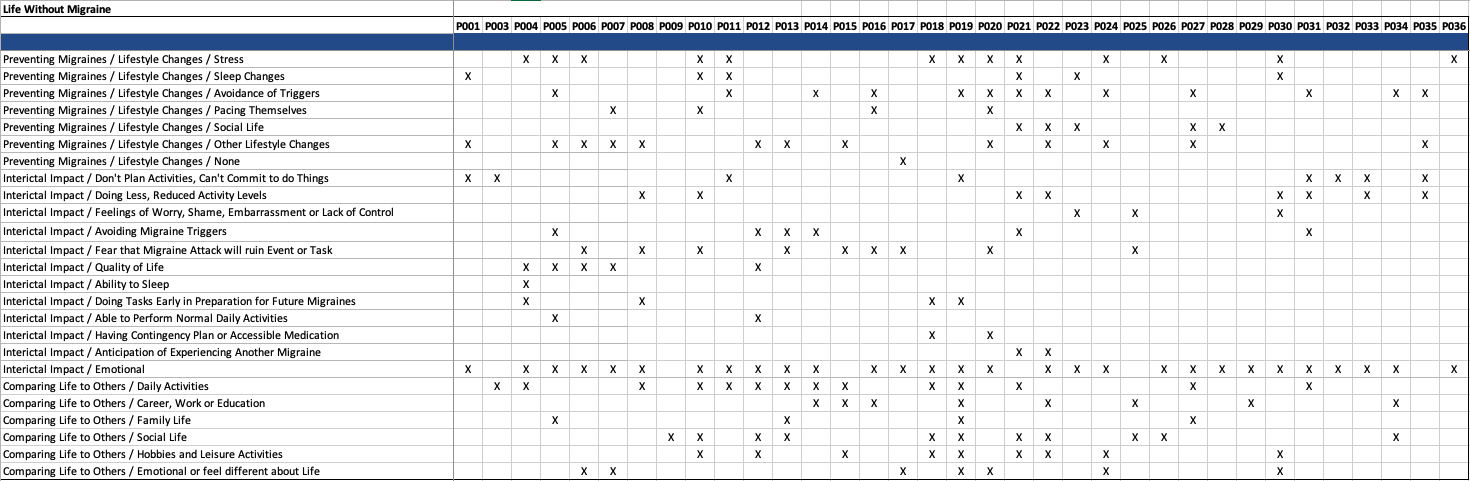


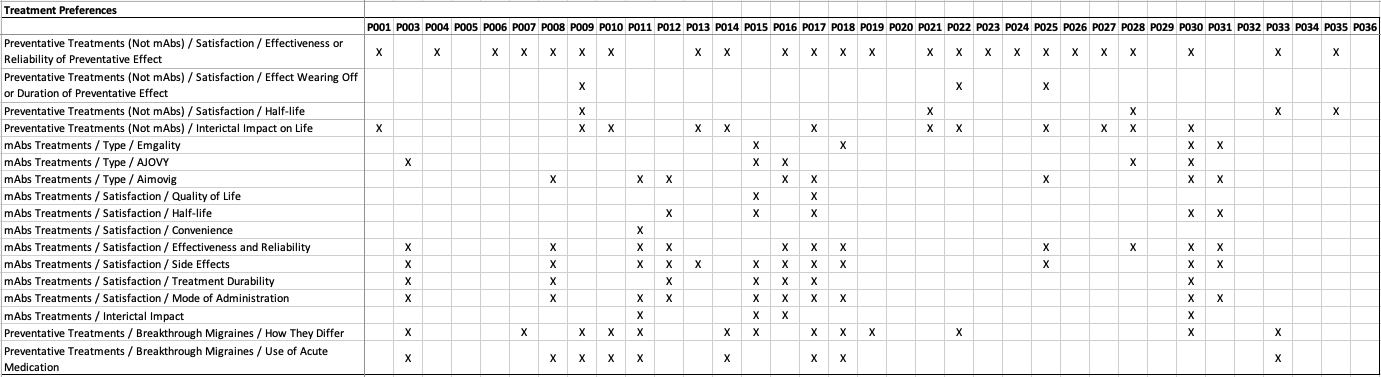

Supplement: Supplementary file 3 — Additional file 3: Saturation tables. The saturation tables document the individuals that identify a certain concept or category. [file 10194_2022_1429_MOESM3_ESM.docx]
